# Beauvericin production by endophytic and epiphytic *Beauveria bassiana* in peach (*Prunus persica*) and implications for insect biocontrol

**DOI:** 10.3389/ffunb.2025.1714008

**Published:** 2025-11-27

**Authors:** Sabrina A. Elgar, Caterina Villari, Conor G. Fair, David I. Shapiro-Ilan, Dario Chavez, Brett R. Blaauw

**Affiliations:** 1 Department of Entomology, University of Georgia, Athens, GA, United States; 2 Warnell School of Forestry & Natural Resources, University of Georgia, Athens, GA, United States; 3 Applied Statistics, College of Agricultural and Environmental Sciences, University of Georgia, Griffin, GA, United States; 4 SE Fruit and Tree Nut Research Laboratory, USDA-ARS, Byron, GA, United States; 5 Department of Horticulture, University of Georgia, Griffin, GA, United States; 6 Institute of Plant Breeding, Genetics, and Genomics, University of Georgia, Griffin, GA, United States; 7 Plant and Environmental Sciences Department, Clemson University, Clemson, SC, United States

**Keywords:** bioinsecticide, biological control, antibiotic, integrated pest management, yellow mealworm, insecticide, mycotoxin

## Abstract

*Beauveria bassiana* (Balsamo) Vuillemin is a well-known entomopathogenic fungus that occupies diverse ecological niches, including soilborne, epiphytic, and endophytic habitats. Its capacity to function as an endophyte has received growing interest in potential applications for sustainable pest management, particularly in woody perennial systems where delivery and persistence of biological control agents are challenging. This study investigated endophytic colonization of peach (*Prunus persica* Batsch) seedlings by *B. bassiana* and quantified production of the insecticidal secondary metabolite beauvericin (BEA) in and on plant tissues. Seedlings were inoculated via foliar spray or soil drench. Fungal recovery was assessed from leaf, stem, and root tissues. Colonization patterns indicated systemic movement, however foliar spray increased recovery from leaf tissues and soil drench increased recovery from roots over time. BEA concentrations varied significantly by tissue type, inoculation method, and surface sterilization status. The highest levels were detected in non-surface-sterilized leaves of foliar-sprayed plants, measured two weeks post-inoculation. Surface sterilization prior to extraction significantly reduced detected concentrations, suggesting that BEA is primarily produced by epiphytic fungal growth. Larval bioassays with *Tenebrio molitor* L. revealed increased mortality associated with foliar-sprayed tissues, aligning with observed BEA levels and suggesting localized insecticidal activity. These findings demonstrate that the spatial dynamics of fungal colonization and metabolite localization are critical considerations for the effective deployment of *B. bassiana* in biocontrol strategies. Further research is needed to determine how environmental factors, host physiology, fungal strain, and time influence secondary metabolite production in and on plants treated with *B. bassiana*.

## 1 Introduction

Peach [*Prunus persica* (L.) Batsch] is a culturally and economically important fruit crop in the southeastern United States, with Georgia and South Carolina ranking among the top producers nationally (NASS, 2024). However, the region’s warm, humid climate supports a diverse array of insect pests and pathogens that jeopardize tree health and fruit production. Management of key pests, particularly cryptic borers, relies heavily on calendar-based applications of broad-spectrum insecticides like chlorpyrifos (Blaauw et al., 2025). While effective in the short term, these chemicals pose substantial risks including resistance development, environmental contamination, regulatory restriction, and negative impacts on non-target organisms (Lacey and Shapiro-Ilan, 2008; Foong et al., 2020; Nandi et al., 2022; Wołejko et al., 2022). These challenges underscore the urgent need for sustainable alternatives such as biological control agents that can be incorporated into integrated pest management (IPM) programs.

*Beauveria bassiana* (Balsamo) Vuillemin (Ascomycota: Hypocreales) is a widely studied, commercially available entomopathogenic fungus (EPF) used for the biological control of many insect pests and plant pathogens (Faria and Wraight, 2007; Ownley et al., 2010; Ferus et al., 2019; Bamisile et al., 2021a; Sharma et al., 2023);. Traditionally applied to plants and substrates as a foliar spray or soil treatment, *B. bassiana* infects insects through cuticle penetration, and performs its entomopathogenic activity through internal colonization, and ultimately host mortality, mediated in part by the production of insecticidal metabolites (Mascarin and Jaronski, 2016).

In addition to colonizing insects, *B. bassiana* can colonize plant surfaces as an epiphyte, as well as internal tissues, as an endophyte. In this latter case, *B. bassiana* lives asymptomatically within different tissues of the plant and has been observed to offer a variety of benefits, including plant growth promotion and pest suppression (Ownley et al., 2010; Vega, 2018; Jaber, 2018; Espinoza et al., 2019; Mantzoukas and Eliopoulos, 2020; Bamisile et al., 2021b; Sui et al., 2023; Vega, 2008). As an insect biocontrol tactic, the endophytic presence of *B. bassiana* has been associated with reduced insect development, fecundity, and survival, offering the potential to target pests within plant tissues (Cherry et al., 2004; Powell et al., 2009; Garrido-Jurado et al., 2017; Rondot and Reineke, 2018; Russo et al., 2019; Barra-Bucarei et al., 2020; Ramakuwela et al., 2020; Shin et al., 2022; Darsouei et al., 2024; Barta, 2018). Furthermore, the endophytic state may improve fungal persistence under abiotic stressors such as UV radiation, high temperature, and desiccation (Mishra et al., 2015; Acheampong et al., 2020), potentially enhancing its biocontrol efficacy in the field. Despite its promise, however, many aspects of this symbiosis remain poorly understood.

One underexplored dimension is the role of fungal secondary metabolites, such as beauvericin (BEA), in mediating insecticidal effects when *B. bassiana* resides within or on plant tissues. BEA, a cyclic hexadepsipeptide produced by *B. bassiana* and other fungi such as Fusarium and Isaria spp., is increasingly recognized as a potent insecticidal secondary metabolite (Wang and Xu, 2012; Caloni et al., 2020). BEA acts as an ionophore that disrupts cellular ion gradients, leading to cytotoxicity and mortality in diverse insect systems (Grove and Pople, 1980; Calò et al., 2003; Fornelli et al., 2004; Bi et al., 2023). Early bioassays demonstrated ingestion-based toxicity, with mortality of *Aedes aegypti* L. (Diptera: Culicidae) larvae exceeding 80% at 10–20 µg/mL, while adults of *Calliphora erythrocephala* Meigen (Diptera: Calliphoridae) were less sensitive (Grove and Pople, 1980). Subsequent work has shown that BEA impairs insect fecundity and symbiont function: ingestion of 25 µg/mL reduced reproduction and increased embryonic abortion in the aphid *Schizaphis graminum* Rondani (Hemiptera: Aphididae), coinciding with DNA disruption in *Buchnera* bacteriocytes (Ganassi et al., 2002).

In lepidopteran systems, BEA exerts strong cytotoxicity both *in vivo* and *in vitro*. In *Spodoptera frugiperda* Smith (Lepidoptera: Noctuidae) SF-9 cells, cytotoxicity occurred at low micromolar concentrations (IC_50_ ≈ 2.5 µM), with progressive cell death after prolonged exposure (Calò et al., 2003; Fornelli et al., 2004). In *Bombyx mori* L. (Lepidoptera: Bombycidae*)*, injection of BEA (lethal concentration 50% (LC_50_) = 362 µM) caused hemocyte vacuolization, reduced viability, and dysregulated immune encapsulation responses (Bi et al., 2023). Similarly, in *Galleria mellonella* L. (Lepidoptera: Pyralidae), fungal infection yielded hemolymph BEA concentrations of 136 ng/mL, with higher levels associated with increased larval mortality and paralysis (Safavi, 2013). Field-relevant studies also demonstrate insecticidal activity: purified BEA achieved up to 100% mortality of spider mites, *Tetranychus urticae* (Acari: Tetranychidae), in laboratory assays and enhanced strawberry yields under greenhouse conditions (Al Khoury et al., 2019). Furthermore, nano-formulated BEA reduced survival of *Glyphodes pyloalis* Walker (Lepidoptera: Crambidae) larvae with an LC_50_ of 0.918 µg/mL, while also impairing digestive enzyme activity and inducing oxidative stress (Yousefi-Lardeh and Zibaee, 2024). Collectively, these studies establish BEA as a multifunctional metabolite capable of reducing insect survival, reproduction, and immune competence across taxa. However, its production and spatial localization, within or on plant tissues following *B. bassiana* inoculation, have not been quantified. This gap limits our understanding of whether and how fungal metabolites contribute to systemic insecticidal effects in plants treated with *B. bassiana*.

Furthermore, a key limitation to the efficient utilization of *B. bassiana* in IPM programs is the difficulty in determining and tracking its insecticidal status following field application. Unlike chemical insecticides whose residual activity can be measured through established residue assays, the persistence and biological activity of *B. bassiana* after application is challenging to measure, limiting growers’ ability to optimize application timing. Developing reliable indicators of fungal activity would greatly enhance the practical use of *B. bassiana* in IPM programs. Because BEA is an insecticidal secondary metabolite primarily produced by *B. bassiana*, quantifying its presence in plant tissues colonized by the fungus may provide a biochemical marker of fungal persistence and insecticidal potential. Thus, beyond testing whether *B. bassiana* produces BEA endophytically, this study explores whether BEA production could serve as a proxy for monitoring the insecticidal status of *B. bassiana* in applied systems.

Although *B. bassiana* has been successfully introduced into over 25 plant species via seed treatment, foliar spray, or soil drench (Bamisile et al., 2018a; Yerukala et al., 2022), its ability to colonize peach as an endophyte has not been previously confirmed. Likewise, no study has quantified BEA levels within or on the surface of peach tissues inoculated with *B. bassiana*.

The objectives of this study were to: (1) determine whether *B. bassiana* can establish as a fungal endophyte in peach seedlings following foliar spray and soil drench inoculation methods; (2) quantify epiphytic and endophytic BEA concentrations in peach tissues; and (3) evaluate the implications of BEA detection as an indicator of fungal persistence and potential insecticidal activity. This work provides foundational insight into the biochemical interactions between *B. bassiana* and its host plant and highlights a potential method to assess fungal activity and improve the efficiency of *B. bassiana* use in IPM programs.

## 2 Materials and methods

### 2.1 Source of *Beauveria bassiana* inoculum

The *B. bassiana* strain GHA (BotaniGard 22WP) was purchased from Arbico Organics (Oro Valley, AZ USA) and subcultured on potato dextrose agar (PDA; Fisher Scientific, Waltham, MA USA). The fungus was incubated for approximately two weeks at 25°C in the dark, and conidia were harvested by flooding plates in 1000 μL of sterile water, scraping the agar surface with a sterile spatula and filtering the collection liquid through a sterile cheesecloth. Conidial concentrations were determined using a Bright-Line™ Hemacytometer (Hausser Scientific, Horsham, PA USA) and the suspensions adjusted to 1 × 10^8^ conidia mL^− 1^ in sterile distilled water containing 0.05% Silwet L-77 (Fisher Scientific) according to Parsa et al. (2013). For all experiments, conidial viability was evaluated by taking a 100 μL sample of each inoculum, plating it on PDA, and incubating it at 25°C for 24 h in the dark. Germination was then assessed under light microscopy by counting germinated spores from a total of 100 randomly selected conidia. Conidia were deemed to have germinated if the germ tube was at least twice the conidia’s length. Only inoculum with a germination of ≥ 90% was used for experiments. The remaining inoculum was stored in the dark at 4°C until viability was confirmed.

### 2.2 Peach seed preparation

Adapting methods from Ramakuwela et al. (2020), under a flow hood, Guardian^®^ rootstock peach pits (sourced from Clemson University, Clemson, SC USA) were surface sterilized by immersion for two minutes in 0.5% sodium hypochlorite (The Clorox Company, Oakland, CA USA) and two minutes in 70% ethanol (Fisher Scientific) then rinsed three times in sterile distilled water. The success of the sterilization process was evaluated by plating 100 µL of the last rinsing water on PDA media, incubating the plate for 10 days at 25 °C and checking for contaminant growth. If growth was seen, the corresponding pits were removed from the experiment and replaced. Pits were cracked using double-bladed pruning shears, which were flame sterilized between each use. Seeds were then submerged in sterile distilled water to hydrate for five days, changing water daily. Under the flow hood, hydrated seeds were placed in stratifying bags containing sterile perlite moistened with sterile distilled water and left for about three months in the dark at °C to germinate.

### 2.3 Inoculation of peach seedlings with *B. bassiana*

Seventy-five germinated seeds were planted in 15.2 cm X 15.2 cm plastic pots containing steam sterilized propagation media (SunGro^®^ Sunshine Mix #5 Propagation Mix, BFG, Burton, OH USA) and Osmocote^®^ 18-6–12 slow-release fertilizer (ICL Specialty Fertilizers, Dublin, OH USA). Plants were then placed in the greenhouse at 25°C under natural light (RH ~55%) where they were watered via drip irrigation for two minutes every other day until they reached their first true leaf stage (approximately three weeks). Forty-eight hours prior to inoculation plants were no longer watered to enhance the uptake of the fungal inoculum. Two treatments, soil drench with a *B. bassiana* inoculum, foliar spray with a *B. bassiana* inoculum, and a control were tested (25 plants each). For soil drench inoculation, a graduated cylinder was used to apply 150 mL suspension of *B. bassiana* (1 x 10^8^ conidia mL^-1^ containing 0.05% Silwet L-77) to the surface of the soil at the base of each of twenty-five plants. For foliar inoculation, a conventional CO_2_ pressurized backpack sprayer was used (BellSpray LLC – R&D Sprayers, Opelousas, LA USA; TeeJet 8002VD yellow flat spray tip, TeeJet Technologies, Glendale Heights, IL USA) to apply approximately 10 mL of *B. bassiana* inoculum (1 x 10^8^ conidia mL^-1^ containing 0.05% Silwet L-77d) to the surface of leaves to each of twenty-five seedlings. With foliar spray treatments the soil base was left uncovered and fungal inoculum allowed to drip off leaves onto soil to mimic field application. The control consisted of both a 10 mL treatment of 0.05% Silwet L-77 sprayed on the surfaces of leaves as well as a 150 mL of 0.05% Silwet L-77 treatment applied to the surface of the soil, applied to each of the remaining twenty-five seedlings. After inoculation, plants were arranged in a randomized block design and allowed to continue to grow in the greenhouse until subsequent tissue sampling.

### 2.4 Re-isolation of endophytic *B. bassiana* in peach tissues by surface sterilization and tissue culturing

**Figure 1:**
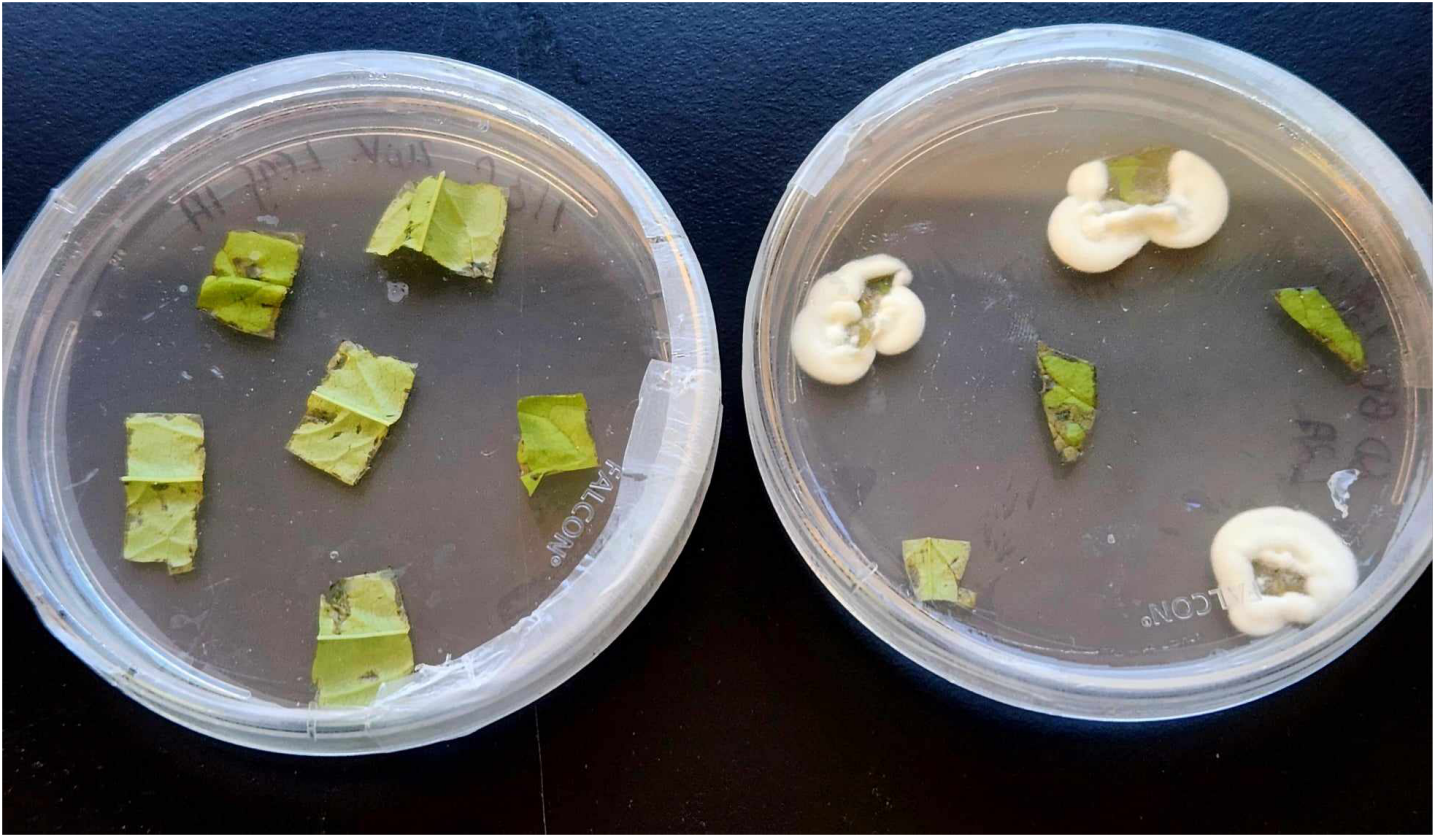
Leaf tissue cultures from *Beauveria bassiana* inoculum soil-drenched (right) and control treated (left) plants. Leaves were sampled two weeks post inoculation (WPI), surface sterilized, plated on DBT media and incubated for ~14 days. Fungi from all plates with tissue samples exhibiting morphological signs of *B. bassiana* (white mycelial growth, left) were re-isolated to fresh media and subjected to subsequent molecular confirmation of *B. bassiana*.

Five peach seedlings per treatment (i.e. treated with *B. bassiana* inoculum via foliar spray, treated with *B. bassiana* inoculum through soil drench) and the control were examined two, four, six, eight-, and twelve-weeks post inoculation (WPI), to confirm endophytic colonization of the fungus. Plants were carefully uprooted and two leaflets, two pieces of the main stem, and two pieces of root were sampled from each plant. Leaves were randomly selected from the middle canopy of the seedling. No leaves were selected from the apical or basal area of the plant. Of the two parts of the main stem, one was sampled towards the middle of the plant and the second closer to the soil surface. The two root sections were obtained by dividing the root system into two parts. Working under a laminar flow hood, all tissue samples were separately surface sterilized to remove epiphytic fungi and surface contaminants as follows: Immersion in 0.5% sodium hypochlorite for thirty seconds, followed by immersion in 70% ethanol for thirty seconds, and rinsing in sterile distilled water three times and success of sterilization was evaluated as previously described. Tissue samples were then allowed to dry on sterile cheesecloths. The outer edges of each tissue sample were trimmed and further cut into six ~ 4 mm X 4mm pieces. Tissue pieces were then plated on selective Doberski and Tribe medium (DBT; Doberski and Tribe, 1980). Cultures were incubated at room temperature in the dark for ~ 14 days. Tissue cultures exhibiting typical mycelial growth of *B. bassiana* (**Figure 1**) were re-isolated onto fresh DBT and allowed to grow for 21 days at room temperature in the dark for subsequent molecular identification.

### 2.5 Molecular validation of fungal re-isolates as *B. bassiana*

The identity of the re-isolated fungal from tissue sample cultures grown on DBT was confirmed via DNA barcoding, including a pure culture of the commercial *B. bassiana* strain GHA as a positive control. DNA was extracted from mycelia collected from each colony using a ZR Fungal/Bacterial DNA MiniPrep Kit (Zymo Research, Irvine, CA USA) following the manufacturer’s protocol. Sterile distilled water was used as a negative control to check for contamination and DNA samples were stored at –20°C until they were processed for PCR.

Following methods similar to Posada and Vega, 2006, the ITS region of the nuclear ribosomal DNA repeat was sequenced for each re-isolate utilizing the primers ITS1-F (fungal-specific) (Gardes & Bruns, 1993) and ITS4 (White et al., 1990). PCRs were performed in 25 μL reaction volumes with 12.5 μL GoTaq Green Master Mix (Promega, Madison, WI USA), 1 μL each of 10 mM primers, and 1 μL of 10 ng/μL DNA or 1 μL of a sterile water control. Amplification was achieved with an initial denaturation step of 5 min at 94°C; 35 cycles of 30 s at 94°C, 45 s at 50°C, and 45 s at 72°C; and a final extension of 7 min at 72°C. PCR products were run on an agarose gel, stained with SYBR Safe (Fisher Scientific), and viewed under UV light. An All-Purpose Hi-Lo DNA Marker (Bio-nexus, Oakland CA USA) was used to visually estimate amplicon size. Samples with amplicons of the correct size (~464 bp) were sent to Eurofins Genomics for Sanger sequencing. Sequencing results were trimmed as needed and contiguous sequences for each isolate were assembled in Geneious Prime version 2024.0.3 (www.geneious.com). Sequences were compared to those of the reference *B. bassiana* strain using the BLAST tool on GenBank (National Center for Biotechnology Information, National Institute for Health, Bethesda, MD USA) to confirm identity. Based on this molecular validation, tissue sample plates (leaf, stem, or root) resulting in positive *B. bassiana* fungal re-isolates were recorded as 1 and those not containing *B. bassiana* fungal re-isolates were recorded as 0.

### 2.6 Quantification of epiphytic and endophytic BEA

#### 2.6.1 Plant material and sample preparation

A total of thirty seedlings were germinated, planted, and grown in the greenhouse as previously described. Ten plants were treated with the fungal inoculum via foliar spray and ten plants were treated with the fungal inoculum via soil drench. The ten remaining plants were prepared as controls. At two WPI, six leaves, six pieces of stem, and six roots were collected from each plant. The first set of two leaves, two pieces of stem, and two roots from each plant were immediately frozen in liquid nitrogen. The second set of two leaves, two pieces of stem, and two root samples from each plant were surface sterilized separately by immersion in 0.5% sodium hypochlorite for thirty seconds, followed by immersion in 70% ethanol for thirty seconds, and rinsing in sterile distilled water three times, prior to freezing in liquid nitrogen. This surface sterilization was performed to remove epiphytic fungi, enabling comparison of BEA concentrations associated with external (epiphytic) versus internal (endophytic) fungal presence. The success of sterilization was evaluated by plating 100 µL of the last rinsing water on PDA media, incubating the plate for 10 days at 25 °C and checking for contaminant growth. If growth was seen these samples were removed from the experiment and replaced with a new collection from additionally treated plants. The remaining tissue samples were also subjected to surface sterilization, re-isolation and molecular confirmation of *B. bassiana* following the same protocol as previously described to confirm successful endophytic colonization of the fungus. The entire experiment was conducted twice.

All tissue samples frozen in liquid nitrogen were then freeze-dried (FreeZone 2.5 Liter Benchtop Freeze Dry System, Cat no. 7670520; Labconco, Kansas City, MO USA) and processed immediately. The tissue was ground into powder using a tissue homogenizer (SPEX SamplePrep 1600 MiniG; Cole-Parmer SamplePrep, Metuchen,NJ USA) and 10 mg were weighed into 1.7 mL microcentrifuge tubes. The extraction procedure was conducted following the protocol by Rämö et al., 2021. Briefly, 250 uL of extraction solvent [3:1 methanol: water, v/v; (LC-MS grade; Sigma-Aldrich, Burlington, MA, USA)] were added to the tissue with 100 ng of isotopically labeled cinnamic acid-13C_3_ (Cat. No. 513962; Sigma-Aldrich) in each sample as the internal standard. Samples were then sonicated and vortexed for 15 min in ice-cold water and centrifuged (5 min at 4°C, at 15,000 rpm), the supernatant was transferred to a new microcentrifuge tube, and the pellet re-extracted with 250 uL of extraction solvent. All supernatants were pooled, filtered through 0.22um PTFE filters (Agilent Technologies, Santa Clara, CA USA, Cat no. 5191-5912), concentrated using a nitrogen stream, re-suspended in the extraction buffer, and stored at -80 °C until further analysis.

#### 2.6.2 UHPLC-ESI-MS analysis

BEA was quantified using reverse-phase ultra-high performance liquid chromatography-electrospray ionization-mass spectrometry (UHPLC-ESI-MS), as described in detail by Rämö et al., 2021. Analyses were performed on an Agilent 1290 Infinity II UHPLC system with autosampler and high-speed pump (Agilent Technologies, Santa Clara, CA, USA) coupled to an Agilent 6546 LC/QTOF mass spectrometer (Agilent Technologies). Chromatographic separation was achieved on a Zorbax Eclipse Plus C18 column (2.1 x 50 mm, 1.8 μm; Agilent Technologies, Cat. No 959757-902) maintained at 40 °C. Mobile phase A was water with 0.1% formic acid (v/v; Fisher Scientific, Waltham, MA, USA) and mobile phase B was acetonitrile with 0.1% formic acid (v/v; Sigma-Aldrich). The flow rate was 0.50 mL min^-1^, and the injection volume was 1 μL. The gradient was: 3% B from 0.0-2.5 min, increased to 15% B at 4.0 min, to 30% B at 6.0 min, to 50% B at 8.0 min, and to 100% B at 10.0 min, held at 100% B until 12.0 min, followed by return to initial conditions and re-equilibrium. Mass spectrometric detection was in positive ESI mode with nitrogen gas temperature 250 °C, nebulizer pressure 40 psi, and capillary voltage 3,000 V. Data were acquired in MS mode and processed using Agilent MassHunter Workstation (Qualitative Analysis, v10.0). Quality control procedures included solvent blanks, calibration check standards, and system suitability standards at the start of the sequence and after each analytical batch to monitor retention time stability, sensitivity, and carryover, as described in detail by Rämö et al., 2021.

#### 2.6.3 Standard curve analysis

Quantification was performed against a calibration curve prepared from an authentic BEA standard (≥97% HPLC purity, Cat. No. B7510; Sigma-Aldrich) in the extraction solvent mixture as described by Rämö et al., 2021. The standard curve consisted of 10 points (1 μL working solutions ranging from 0.1–10 ng μL^-1^) prepared in methanol (LC-MS grade; Sigma-Aldrich) containing the same concentration of isotopically labeled internal standard, cinnamic acid-13C_3_ (Cat. No. 513962; Sigma-Aldrich), as used for samples. Each standard and sample were analyzed in triplicate injections to determine concentration. The calibration curve showed an R^2^ of 0.981, generated using Agilent MassHunter Workstation (Quantitative Analysis v11.0).

#### 2.6.4 Limit of BEA detection and quantification

The limit of detection (LOD) and limit of quantification (LOQ) were used to evaluate method sensitivity. The criteria for a detectable signal included correct retention time, a sufficient signal-to-noise ratio (S/N), and presence of confirmation ions for BEA. LOD was calculated as the average peak area plus three times the standard deviation (SD), and LOQ as the average peak area plus six times the SD (Rämö et al., 2021). In addition, as an alternative definition, LOD and LOQ may be expressed as the lowest concentrations at which the analyte produced a peak signal three- and tenfold higher than the background noise, respectively (Yogendrarajah et al., 2013). Using this approach, the method LOD and LOQ for BEA were determined to be 1.950 ng g^-1^ and 5.910 ng g^-1^, respectively. We considered samples with resulting BEA concentrations that fell between LOD and LOQ to contain trace amounts of the mycotoxin and samples containing values below the LOD to contain none of the mycotoxin.

### 2.7 *Insect* leaf feeding assay

To evaluate the functional significance of BEA detection as an indicator of fungal persistence and potential insecticidal activity, a total of thirty seedlings were germinated, planted, and grown in the greenhouse as previously described. Ten plants were treated with the fungal inoculum via foliar spray and ten plants were treated with the fungal inoculum via soil drench. The ten remaining plants were prepared as controls. Two WPI, six leaves of around the same size were collected from each plant to be utilized for feeding assays and confirmation of endophytic presence of *B. bassiana*. Of the collected leaves, two leaves from each plant were surface sterilized separately by immersion in 0.5% sodium hypochlorite for thirty seconds, followed by immersion in 70% ethanol for thirty seconds, and rinsing in sterile distilled water three times to remove epiphytic fungi, enabling comparison of feeding assay outcomes and BEA exposure between epiphytic and endophytic fungal associations, while the other two leaves were not surface sterilized prior to use in feeding assays. The success of the sterilization procedure was evaluated by plating 100 µL of the last rinsing water on PDA media, incubating the plate for 10 days at 25°C, and checking for contaminant growth. If growth was seen, these samples were removed from the experiment and replaced with new samples from additionally treated plants. The remaining two leaf samples per plant were subjected to re-isolation and molecular confirmation of *B. bassiana* as previously described, to confirm the endophytic presence of the fungus.

Leaves collected and prepared for feeding assays were placed in an individual feeding chamber, which consisted of a 100 mm Petri dish (Fisher Scientific), lined with 9.0 cm filter paper (Fisher Scientific) moistened with 1000 µL of sterile distilled water. One third instar yellow mealworm, *Tenebrio molitor* L. (Coleoptera: Tenebrionidae) larva, (Fluker Farms, Port Allen, LA, USA) was added to each chamber and the dish sealed with parafilm. Although *T. molitor* is not an insect pest of peach this species was chosen as a model in our study due to its generalist feeding behavior, ease of rearing, and potential to replicate these experiments in other plant systems. The feeding chambers were then incubated at 26°C and mortality was assessed and recorded daily for 21 days. Larvae were considered dead if they failed to respond to gentle prodding. Twenty larval feeding chambers were created per foliar spray, soil drench, and control treatment for the experiment and the entire experiment was conducted twice.

### 2.8 Statistical analysis

All statistical analyses and data visualization were performed in R version [4.3.2] (R Core Team, 2023) using base functions and relevant packages.

#### 2.8.1 Assessment of *B. bassiana* as an endophyte in peach

To test the hypothesis that the probability of observing the presence of *B. bassiana* as an endophyte would depend on the continuous variable of Time (WPI), three levels of Treatment (foliar spray, soil drench, or control), and three levels of Tissue (leaf, root, stem), as well as the interaction between Treatment and Tissue, we fit a generalized linear mixed model with a binomial distribution and a logit link function using the glmer function in the *lme4* package (Bates et al., 2014). To account for repeated measurements from the same individual plant, we included plant identity as a random intercept. Following the fitting of the model, the simulateResiduals function from the *DHARMa* package (Hartig, 2024) was used to assess the distribution of residuals and confirm that the model assumptions (e.g., normally distributed residuals, and homogeneity of variance, etc.) were met. Model significance was assessed with the Anova () function in the *car* package (Fox and Weisberg, 2019) and *post hoc* comparisons were conducted via the *emmeans* package (Lenth, 2025) using Sidak’s test (α = 0.05). Lastly, sample predictions were fit using the predict function in the *stats* package (R Core Team, 2024) and plotted with the raw data for visualization with the ggplot function in the *ggplot2* package (Wickham, 2016).

#### 2.8.2 Quantification of BEA

We recorded samples containing BEA values below the analytical limit of detection (LOD = 1.950 µg/g) as zero. Surface sterilization was excluded from analyses because all sterilized samples were below the LOD and contained no detectable BEA. To test the hypothesis that both the probability of detecting BEA above the LOD and the conditional concentration of BEA would depend on the interaction between the three levels of Treatment (foliar spray, soil drench, or control) and the three levels of Tissue (leaf, root, or stem), we initially fit a linear model with Treatment, Tissue, and their interaction as predictors. However, diagnostic tests indicated strong violations of assumptions: residuals were significantly non-normal (Shapiro–Wilk p < 0.001) and heteroscedastic (Breusch–Pagan test p < 0.001). A Poisson generalized linear model was also evaluated but proved unsuitable due to extreme overdispersion (dispersion ratio ≈ 201, p < 0.001) and significant zero inflation (p < 0.001; DHARMa residual diagnostics via the *DHARMa* package, Hartig, 2024).

Given these results, the data were more appropriately modeled with a two-part hurdle approach, which accommodates both excess zeros and skewed positive values (Zuur et al., 2009; Brooks et al., 2017). The first component was a binary logistic regression modeling the probability of detecting BEA above the LOD (BEA > 1.950 µg/g). The second component was a Gamma generalized linear model with a log link, fit only to samples with positive concentrations, to estimate conditional mean levels of BEA. Both models included Treatment, Tissue, and their interaction as fixed effects and were fit utilizing the *glmmTMB* package in R (Brooks et al., 2017). Residual diagnostics from the *DHARMa* package (Hartig, 2024) confirmed that the hurdle models exhibited no overdispersion, residual bias, or outliers. Model significance was assessed with the Anova () function via the *car* package (Fox and Weisberg, 2019) and *post hoc* comparisons were conducted via the *emmeans* package (Lenth, 2025) using Sidak’s test (α = 0.05). To facilitate interpretation, we combined the predicted detection probabilities from the logistic component (p) with the conditional mean concentrations from the Gamma component (μ_+_). Overall expected concentrations were calculated as:


$$\text{Ε}[BEA]=p\;\times \;\mu_{+}$$

In plain terms, this value is the probability of detecting BEA multiplied by the average concentration among those samples where it was detected, yielding an overall expected mean that represents the average concentration across all samples, including both non-detects and positives.

#### 2.8.3 Assessment of *T. molitor* mortality

To test the hypothesis of a two-way interaction between the three levels of *B. bassiana* Treatment (foliar spray, soil drench, control) and the two levels of Surface Sterilization (yes or no) on the probability of yellow mealworm mortality, we used a generalized linear mixed model with a binomial distribution and logit link function using the glmer() function in the *lme4* package (Bates et al., 2014). The probability of yellow mealworm mortality was the dependent variable and the two-way interaction of treatment and surface sterilization, and main effects were the independent variables. The experimental trial was designated as a random effect. Following the fitting of the model, the simulateResiduals function from the *DHARMa* package (Hartig, 2024) was used to assess the distribution of residuals to determine if the model assumptions (e.g. normally distributed residuals, and homogeneity of variance, etc.) were met. Model significance was assessed using the Anova () function in the *car* package (Fox and Weisberg, 2019). *Post hoc* comparisons were conducted via the *emmeans* package (Lenth, 2025) using Sidak’s test (α = 0.05) nesting treatment within surface sterilization status and plotted for visualization with the ggplot function in the *ggplot2* package (Wickham, 2016).

## 3 Results

### 3.1 Systemic endophytic colonization of *B. bassiana* in peach

**Figure 2:**
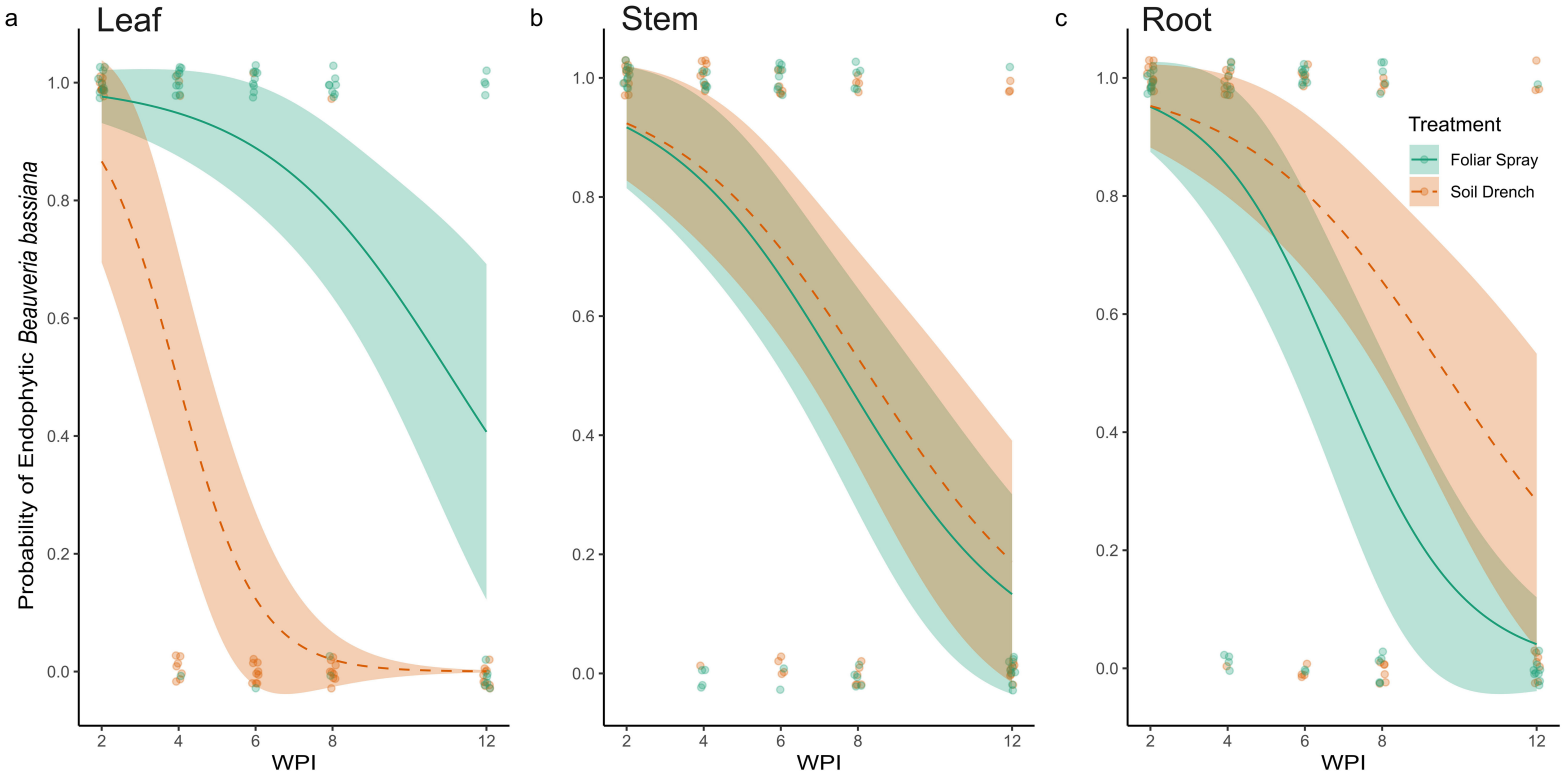
Probability of the presence of endophytic *Beauveria bassiana* in leaves **(A)**, stem **(B)**, and root **(C)** tissues of plants foliar sprayed (green) and soil drenched (orange) with fungal inoculum two to twelve weeks post inoculation (WPI). No control samples tested positive for the presence of *B. bassiana*, therefore are not represented in the figure.

Results from fungal re-isolation via tissue culturing indicate that the entomopathogenic fungus *B. bassiana* can be successfully established as an endophyte in peach seedlings using both foliar spray and soil drench inoculation methods (**Figure 2**). The PCR amplification of fungal re-isolates was consistent in generating an amplicon of the appropriate size (~464 bp), specific to *B. bassiana*. Sequencing confirmed the identity of amplified fragments as *B. bassiana* and pairwise alignment against previously characterized sequences of *B. bassiana* on GenBank (Accession number: PP318546) was recorded for all positive samples. The main effects of Treatment (χ² = 23.72, df = 1, p < 0.001), Tissue type (χ² = 6.17, df = 2, p = 0.046), and Time (WPI; χ² = 33.17, df = 1, p < 0.001) significantly affected the probability of recovery of *B. bassiana* as an endophyte from peach seedlings. In addition, a significant Treatment × Tissue interaction was detected (χ² = 27.59, df = 2, p < 0.001), indicating that the effect of treatment varied among plant tissues.

**Figure 3:**
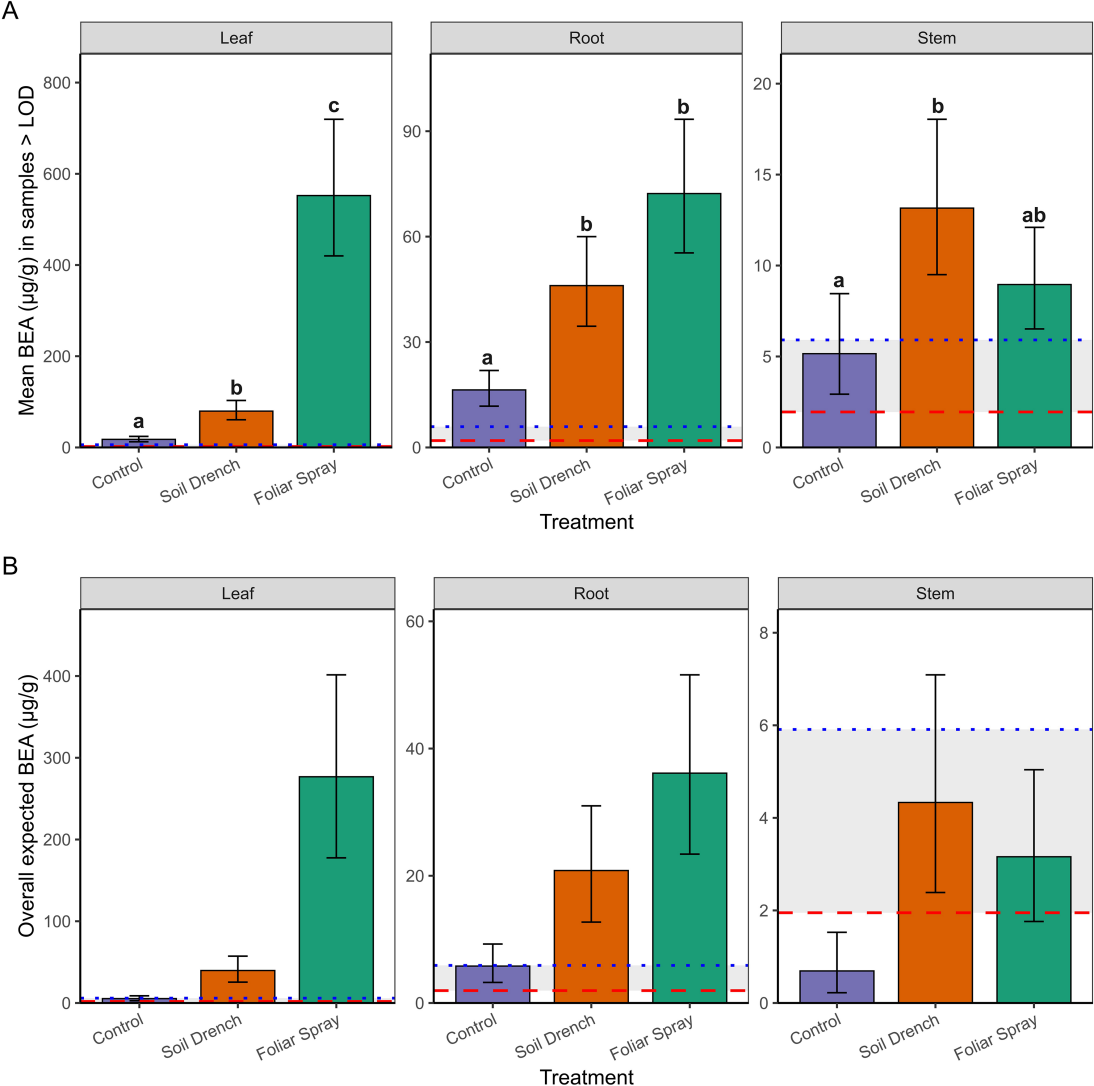
Concentrations of beauvericin (BEA; µg/g tissue) detected in peach seedlings following inoculation with *Beauveria bassiana* strain GHA. **(A)** Mean concentrations (± SE) among samples with detectable BEA (>LOD = 1.95 µg/g; >LOQ = 5.91 µg/g) across treatments (Control, Soil Drench, Foliar Spray) and tissues (Leaf, Root, Stem). **(B)** Overall expected concentrations (± SE), which include zero values for samples without detectable BEA, thereby representing treatment and tissue-specific means on a per-sample basis. Red dashed line indicates the LOD threshold and blue dashed line indicates the LOQ threshold. The same letters indicate no significant between mean beauvericin concentrations (Sidak’s test, a = 0.05).

At two WPI, all sampled tissues (leaves, stems, and roots) from both foliar-sprayed and soil-drenched plants tested positive for endophytic colonization by *B. bassiana* (**Figures 2**, **3**). We recognized this as an opportunity to investigate what occurs metabolically with BEA when *B. bassiana* can be detected systemically, therefore all other experiments in our study were conducted 2 WPI. Over time, the probability of recovering *B. bassiana* from leaf tissues cultures was higher in foliar-sprayed plants compared to soil-drenched plants (**Figure 2A**). In soil-drenched plants, recovery from leaves declined sharply around six WPI and ceased entirely by twelve weeks (**Figure 2A**). In contrast, recovery from stem tissues was similar between foliar spray and soil drench treatments throughout the study period (**Figure 2B**). By eight WPI, the likelihood of recovery from stems was approximately 50%, regardless of treatment method (**Figure 2B**). For root tissues, the probability of endophytic recovery was slightly higher in soil-drenched plants compared to those treated via foliar spray (**Figure 2C**). Overall, the probability of recovering *B. bassiana* from all tissue types declined over time. However, in foliar-sprayed plants, the fungus was still detectable in representative samples of all tissue types at twelve WPI (**Figure 2**). There was no recovery of *B. bassiana* in any of the control plants.

### 3.2 Quantification of BEA

Surface sterilized tissues consistently contained BEA concentrations below the limit of detection (LOD = 1.95 µg/g), indicating that in this study measurable BEA production was only present epiphytically. Therefore, analyses were restricted to non-sterilized tissues to evaluate treatment effects on epiphytic BEA production.

To do so we used a two-part hurdle modeling framework. The first component modeled the probability of detecting BEA above the limit of detection (LOD = 1.95 µg/g) in non-surface sterilized tissues. Detection probability did not differ significantly among Treatments (foliar spray, soil drench, control), Tissue (leaf, root, stem), or their interaction (Anova, χ² = 4.25, df = 2, p = 0.12 for Treatment; χ² = 5.41, df = 2, p = 0.07 for Tissue; χ² = 1.39, df = 4, p = 0.85 for Treatment × Tissue; **Supplementary Figure S1**). Because detectability did not differ among treatments or tissues, subsequent differences reflect true variation in concentrations rather than artifacts of threshold sensitivity.

The second component of the analysis estimated mean concentrations conditionally among samples with detectable BEA (BEA >LOD; **Figure 3A**). Treatment and tissue type as well as the interaction between treatment and tissue type significantly influenced BEA concentrations in non-surface sterilized peach tissue samples. Foliar spray inoculation consistently yielded the highest concentrations, especially in leaves (mean: 549 µg/g; 95% CI: 422–714 µg/g, range: 127 – 1517 µg/g), which were significantly greater than both soil drench and control treatments (p < 0.0001). Roots also exhibited elevated concentrations under foliar spray (71 µg/g; 95% CI: 55–93 µg/g, range: 27.7-119 µg/g) compared to controls (p < 0.0001). Stems contained lower concentrations overall (≤18 µg/g), with only the soil drench treatment producing levels significantly higher than the control (p = 0.0068).

Finally, by combining detection probability with conditional means, we derived overall expected BEA concentrations (i.e., the average amount expected across all samples, including non-detects; **Figure 3B**). This metric revealed the strongest treatment effects in leaves, where foliar spray application produced mean expected concentrations nearly an order of magnitude greater than soil drench and two orders of magnitude greater than controls. Roots showed intermediate effects, while stems exhibited only trace to low expected concentrations. Notably, concentrations between the LOD (1.95 µg/g) and LOQ (5.91 µg/g) were considered trace, and such levels were observed primarily in control and stem tissues. Together, these results demonstrate that foliar spray inoculation is the most effective method for achieving high BEA concentrations in peach seedlings, with the strongest accumulation on leaves. Soil drench application yielded intermediate concentrations, whereas stems rarely accumulated levels above trace amounts.

### 3.3 *T. molitor* mortality coincides with BEA Production

**Figure 4:**
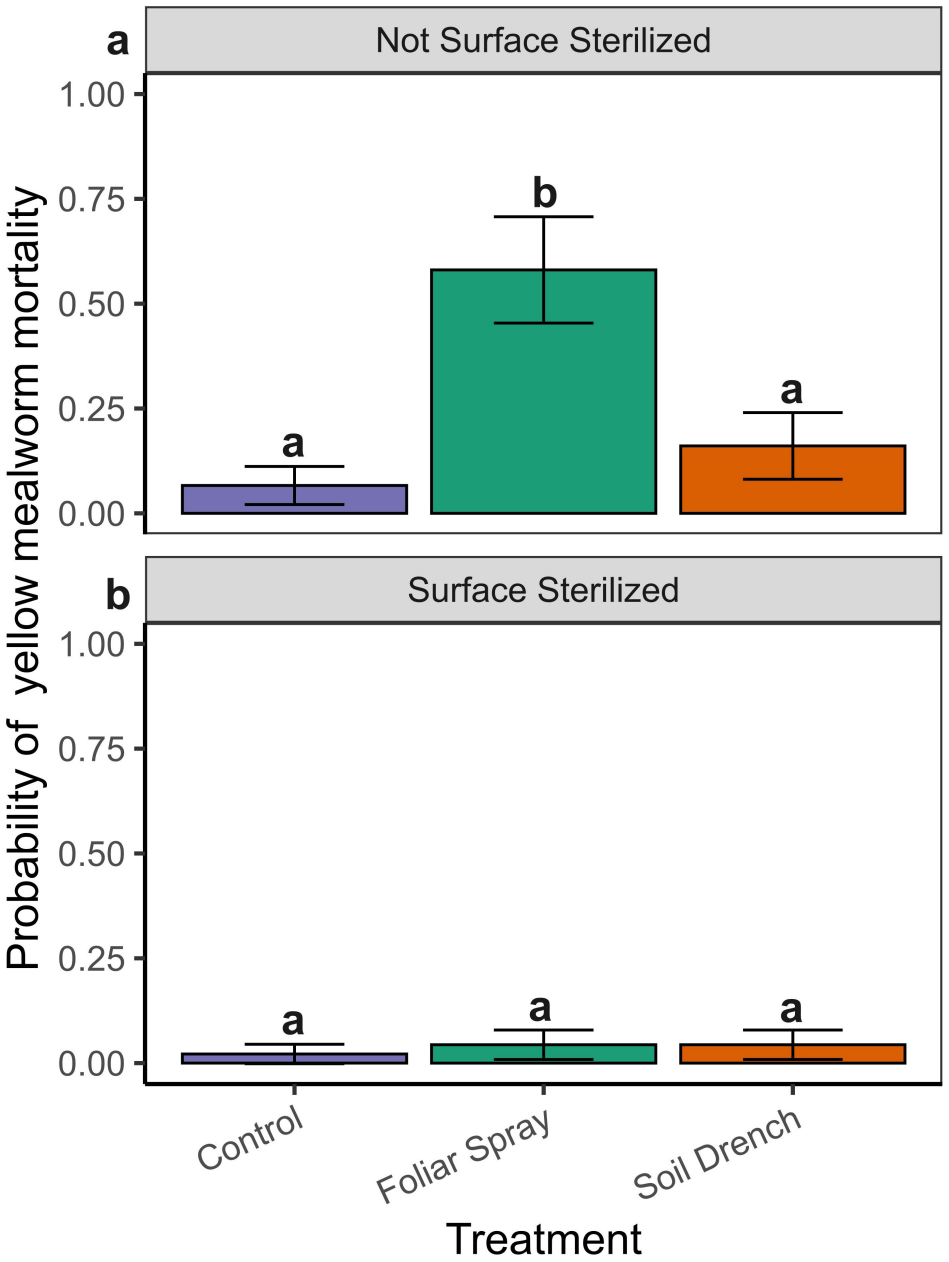
Probability (mean ± SE) of yellow mealworm (*Tenebrio molitor*) mortality exposed to non-surface sterilized **(A)** and surface sterilized **(B)** leaf tissues sampled from plants treated with a control, soil-drenched, or foliar-sprayed with a *Beauveria bassiana* inoculum. The same letters indicate no difference in the probability of mealworm mortality between treatments under each surface sterilization status (Sidak’s test, a = 0.05).

*Tenebrio molitor* feeding assays revealed significant main effects of treatment (*x^2^* = 18.33, df = 2, P = < 0.001) and surface sterilization status (*x^2^* = 19.40, df = 1, P = < 0.0001) on yellow mealworm mortality. However, the interaction between treatment and sterilization was not statistically significant (*x^2^* = 3.04, df = 2, P = 0.219), suggesting that treatment and surface sterilization independently influenced mortality. *Post hoc* comparisons within each sterilization group clarified these effects. In the non-surface sterilized group, yellow mealworm mortality was significantly higher when exposed to leaf tissue foliar sprayed with the fungus (P < 0.05; *prob* = 0.578 ± 0.106 SE) compared to both control (*prob* = 0.118 ± 0.059 SE) and soil drench (*prob* = 0.167 ± 0.072 SE) treatments (**Figure 4A**). No significant difference in mortality was observed between control and soil drench treatments in this group. In contrast, when leaves were surface sterilized yellow mealworm mortality was uniformly low across all treatments (*prob range:* 0.023 – 0.047), with no significant differences detected among control, foliar spray, or soil drench treatments (**Figure 4B**).

## 4 Discussion

This study demonstrates that endophytic colonization of peach seedlings by *B. bassiana* strain GHA is feasible, thereby expanding the range of woody perennials – currently including pecan (*Carya illinoinensis* Koch), pine (*Pinus radiata* D. Don), date palm (*Phoenix dactylifera* L.), cacao (*Theobroma cacao* L.), horse chestnut (*Aesculus hippocastanum* L.), cassava (*Manihot esculenta* Crantz), and coffee (*Coffea arabica* L.) - known to support artificial endophytic establishment of this fungus (Posada and Vega, 2005, 2006; Brownbridge et al., 2012; Barta, 2008; Greenfield et al., 2016; Ramakuwela et al., 2020; Husain et al., 2024). Our findings indicate that peach seedlings are highly receptive to colonization, with both soil drench and foliar spray inoculation methods resulting in successful recovery of *B. bassiana* from all sampled leaf, main stem, and root tissues two WPI. Re-isolation of the fungus from tissues distal to the inoculation sites, such as leaf tissues from soil-drenched plants, suggests systemic movement and vertical transmission within the host. As in other plant hosts, endophytic colonization of peach by *B. bassiana* was transient, with a general decline in fungal recovery over time and patterns of persistence varied by inoculation method and tissue type (Garrido-Jurado et al., 2017; Bamisile et al., 2018b; Vega, 2018; Ramakuwela et al., 2020). By 12 WPI, the probability of fungal recovery had dropped below 50% across all treatments. Notably, the foliar spray treatment was associated with a higher probability of re-isolation from leaf tissues, while the soil drench treatment showed slightly greater persistence in root tissues over time. These differences suggest that the method of inoculation may influence the longevity and distribution of *B. bassiana* colonization within specific peach tissues. Together, these results highlight the potential of *B. bassiana* to establish systemic transient endophytic associations in peach seedlings and underscore the importance of considering inoculation method, timing, and repeated application when aiming to establish this fungus as an endophyte in specific tissue types and time periods.

A central novel finding of this study is the quantification of beauvericin (BEA), a cyclic hexadepsipeptide with insecticidal, antimicrobial, and cytotoxic properties (Caloni et al., 2020), in peach tissues following *B. bassiana* inoculation. Our results demonstrate that measurable BEA accumulation occurred primarily in non-surface-sterilized tissues, suggesting that epiphytic colonization and surface-associated fungal growth contribute most strongly to its production under greenhouse conditions. The highest concentrations were detected in leaves from foliar-sprayed plants, averaging 549 µg/g tissue (95% CI: 422–714 µg/g), with maximum levels reaching 1,517 µg/g. Roots also accumulated moderate concentrations (mean 71 µg/g), while stems contained comparatively low amounts (≤18 µg/g).

These values are biologically meaningful. The mean concentrations on foliar-sprayed leaves (549 µg/g; range 127–1517 µg/g) overlap with or exceed thresholds previously shown to negatively affect insects. For example, ingestion of 1–5 µg/g impaired aphid reproduction (Ganassi et al., 2002), while 10–20 µg/mL caused >80% mosquito larval mortality (Grove and Pople, 1980). Thus, the concentrations observed here fall within biologically active ranges known to reduce insect survival and fecundity. Importantly, BEA levels measured here also exceeded concentrations reported from broth culture or conidial suspensions (often <10 ng/mL; Leland et al., 2005; Safavi, 2013), suggesting that phyllosphere conditions such as humidity, nutrient exudates, and microbial interactions may enhance secondary metabolite biosynthesis.

Environmental and host-associated factors are likely to play key roles in regulating BEA production. Temperature, humidity, and time since inoculation have all been shown to affect mycotoxin biosynthesis in entomopathogenic and phytopathogenic fungi (James et al., 1998; Gutierrez-Pozo et al., 2024; Oluwakayode et al., 2024). For instance, *Fusarium* spp. exhibit peak BEA synthesis under specific temperature and humidity regimes (Gutierrez-Pozo et al., 2024), while *B. bassiana* has been shown to adjust metabolite expression in response to host or environmental conditions (James et al., 1998). In addition, plant factors such as exudates, surface microflora, and tissue physiology may influence fungal metabolic activity. The complete absence of detectable BEA in sterilized tissues highlights the importance of spatial context. Epiphytic associations may stimulate metabolite production more strongly than internal colonization under some conditions. Future studies should therefore quantify temporal patterns of BEA and other *B. bassiana* mycotoxins in both epiphytic and endophytic niches, across different environmental conditions and hosts.

Interestingly, low but detectable levels of BEA were also observed in non-surface-sterilized control tissues, especially in roots. Although not directly tested in this study, one possible explanation is the presence of naturally occurring fungi such as *Fusarium* spp., which are known BEA producers and exist as endophytes in peach (Wang and Xu, 2012; Mannai et al., 2018; Gautier et al., 2020). This complicates interpretation and highlights a broader challenge for field-level monitoring: distinguishing BEA produced by inoculated *Beauveria* from background *Fusarium* contamination is agronomically important. Because *Fusarium*-derived BEA can accumulate in agricultural products (Santini et al., 2012; Pietruszka et al., 2023), its occurrence in untreated peach tissues underscores the need for robust metabolite monitoring in systems where *B. bassiana* is utilized as a biological control tactic potentially increasing BEA in the environment where it is applied.

The functional significance of BEA accumulation was supported by our insect bioassays. Non-surface sterilized leaves from foliar-sprayed plants, which contained the highest BEA concentrations, caused significantly higher mortality in *T. molitor* larvae compared to controls or soil-drenched treatments. While this correlation suggests a role for BEA in insecticidal activity, synergistic effects with other fungal metabolites or enzymes cannot be excluded (Mascarin and Jaronski, 2016). However, these results support BEA detection as an indicator of *B. bassiana* persistence and potential insecticidal activity in general. It is important to note that *T. molitor* serves as a generalist model and does not represent the insect pest complex associated with peach. Feeding guilds differ substantially among peach herbivores: phloem-feeding aphids (*Myzus persicae* Sulzer), cell-piercing thrips (*Frankliniella* spp.), and fruit-, xylem- or cambium-feeding borers may encounter BEA at different concentrations and tissue locations than *T. molitor* (Blaauw et al., 2025). Therefore, while our results demonstrate toxicity, future bioassays with peach-relevant pests are needed to determine the significance of *B. bassiana* and BEA-mediated suppression in peach specific pests.

From an applied perspective, foliar inoculation produced higher BEA accumulation than soil drench, especially on leaves, suggesting this method may yield stronger short-term pest suppression. However, given the transient nature of colonization, repeated applications are likely needed to sustain efficacy. In agricultural systems, understanding and managing the variables associated with BEA production is essential to harness the benefits of *B. bassiana* as a biocontrol agent while minimizing unintended risks. The increased addition of a biologically active mycotoxin to agricultural environments necessitates careful consideration in terms of application timing, harvest intervals, post-harvest processes, and regulatory thresholds. While BEA is not currently regulated in most agricultural contexts, its detection and known bioactivities emphasize the need for monitoring protocols, particularly as mycoinsecticides gain traction in sustainable agriculture. BEA has been reported not only for its insecticidal properties but also for its potential pharmaceutical applications, raising concerns about impacts on non-target organisms, soil microbiomes, food safety, and the potential development of antimicrobial resistance (Sood et al., 2017; Gautier et al., 2020; Křížová et al., 2021; Pietruszka et al., 2023; Al Khoury et al., 2024). Given its antibiotic-like activity, there is a pressing need for monitoring BEA and similar fungal metabolites in treated crops, especially as the use of endophytic and epiphytic fungi and mycotoxins expands in IPM and organic farming practices.

The ability of *B. bassiana* to occupy both endophytic and epiphytic niches and produce metabolites such as BEA underscores the ecological flexibility of this fungus. This dual capacity may facilitate persistence in variable environments and enhance interactions with both plant hosts and herbivores. However, it also complicates predictions of biocontrol outcomes, as metabolite production may be shaped by inoculation method, tissue colonization patterns, host physiology, and environmental stressors. Integrating metabolite monitoring with functional outcomes such as pest suppression, plant growth promotion, and shifts in phytochemistry will be essential to fully understand the roles of endophytic *B. bassiana* and other endophytic entomopathogenic fungi in crop systems as well as proper integration into IPM programs. These results provide the first evidence in peach that endophytic colonization can occur without concomitant metabolite accumulation or insecticidal effect. By distinguishing epiphytic from endophytic pathways, this study clarifies why field outcomes with *B. bassiana* are variable and underscores the importance of timing, formulation, and monitoring in deploying entomopathogenic fungi for integrated pest management. Future studies should evaluate colonization and metabolite production in mature orchard trees under field conditions. Assessing persistence, spatial distribution, and ecological impacts at the orchard scale will be critical to determine the long-term feasibility of deploying *B. bassiana* as a biocontrol tool in peach production.

## Data Availability

The raw data supporting the conclusions of this article will be made available by the authors, without undue reservation.
