# Supplementary material for: Beauvericin production by endophytic and epiphytic Beauveria bassiana in peach (Prunus persica) and implications for insect biocontrol
Source: Front Fungal Biol. 2025 Nov 27;6:1714008. doi: 10.3389/ffunb.2025.1714008 (PMC12695560; doi:10.3389/ffunb.2025.1714008)
Supplement: Supplementary file 1 [file DataSheet1.pdf]

Supplementary Material

1 Supplementary Figures

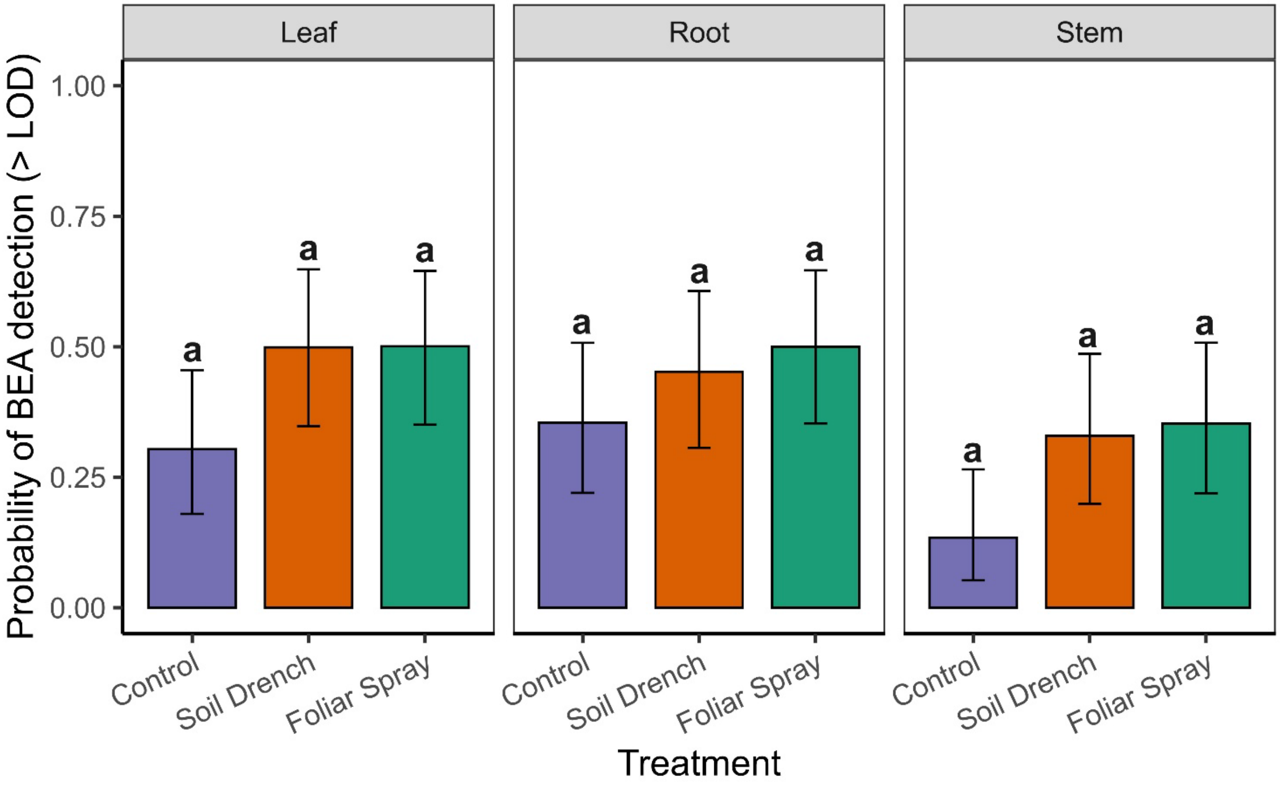

Supplementary Figure 1. Probability of beauvericin (BEA) detection above the limit of detection (LOD; 1.95 µg/g tissue) in non-surface sterilized peach seedling tissues by treatment. Bars represent model-based estimated probabilities (+ 95% CI) of BEA detection. Different letters above indicate pairwise comparisons within each tissue type (Sidak's, $p < 0.05$).
